# CONDUCTING COMMUNITY-BASED RESEARCH AS AN INSIDER AND OUTSIDER IN YOUR OWN COMMUNITY

**DOI:** 10.1093/geroni/igac059.1515

**Published:** 2022-12-20

**Authors:** Jordan Lewis

**Affiliations:** University of Minnesota Medical School, Duluth, Minnesota, United States

## Abstract

As an Alaska Native from the Bristol Bay region, this presentation will discuss being an insider and outsider in your own community and how it impacts research studies. Using case studies from my dissertation research, this presentation will discuss positionality and how to collect objective data, the lack of objectivity in getting, analyzing, and reporting data, and how research methods influenced my study. I will also discuss challenges and benefits of being an insider, including enhancing the validity of the research process, data collection, and analysis, understanding the group’s culture. The results include trusting relationships with communities, the Elders’ voices guiding the findings, an appreciation for the challenge of conducting research as an insider and outsider. This presentation will conclude with a discussion on how to obtain objective data and ensure the accuracy of findings through community-engagement principles and the benefits of working in your own community.

